# Primary health care, access to legal abortion and the notion of ideal victim among medical practitioners: The case of Chile

**DOI:** 10.3389/fpsyg.2022.1007126

**Published:** 2022-11-17

**Authors:** Lidia C. Casas, Juan J. Álvarez, Lieta V. Vivaldi, Adela R. Montero, Natalia Bozo, Jorge Babul

**Affiliations:** ^1^Faculty of Law, Diego Portales University, Santiago, Chile; ^2^Instituto de Filosofía y Humanidades, Universidad Diego Portales, Santiago, Chile; ^3^Faculty of Law, Universidad Alberto Hurtado, Santiago, Chile; ^4^Centro de Medicina Reproductiva y Desarrollo para el adolescente (CEMERA),Facultad de Medicina, Universidad de Chile, Santiago, Chile; ^5^Subsecretaría de la Niñez, Santiago, Chile; ^6^Diego Portales University, Santiago, Chile

**Keywords:** Chile, rape, stereotype, abortion, primary health care, ideal victim

## Abstract

In 2017, Chile enacted new legislation allowing access to legal abortion on three grounds, including rape. This article summarizes a qualitative, exploratory study that examined the role of primary healthcare services in the treatment of rape survivors in order to identify challenges and strengths in accessing legal abortion. The relevant data was collected through 19 semi-structured interviews conducted with key informants. The angry legislative debate that preceded enactment of the 2017 abortion bill evidenced the presence of strong biases against survivors of sexual violence. At the time, abortion opponents sought, *inter alia*, to discredit women who report rape, arguing that such claims would be misused to secure illicit abortions. In actual fact, however, rape has turned out to be the least used of all grounds for abortion, with girls and teens making up the smallest group of seekers. This article presents our findings on rape-related issues, notably the biases and shortcomings of medical practitioners regarding the new abortion law. We noted with concern their failure to screen for sexual violence and propensity to stigmatize the victims, a phenomenon that becomes exacerbated when it involves particularly vulnerable populations, such as girls and women who are poor, homeless, migrant, or who abuse alcohol or drugs. We further noted that prevalent stereotypes based on the notion of the *ideal victim* can revictimize girls and women and work to defeat the intent of the law. In Chile, the primary healthcare system is a key point of entry for abortion. In this highly charged arena, however, lack of political will, compounded by the COVID-19 pandemic, have kept health care practitioners from undergoing timely, gender-sensitive training on the new law, a key requirement for ensuring dignified care and respect for women’s rights. We conclude that if government policy is to prevent multiple, intersectional discrimination, it must recognize the diversity of women and adapt to their specific contexts and singularities.

## Introduction

Law 21030, an Act on the Voluntary Termination of Pregnancy (the VTP Act), was enacted in September 2017. The law amended Health Code Article 119 in order to allow women to terminate pregnancies in cases of grave risk to life, severe lethal fetal impairment, or rape. The angry legislative debate that preceded enactment of the bill evidenced the presence of strong biases against survivors of sexual violence, referred as victims in the law and all the regulations. Abortion opponents sought, *inter alia*, to discredit women who report rape, arguing that such claims would be massively misused to secure illicit abortions. Yet, rape has turned out to be the least used of all three grounds. As official Health Ministry data from January 2018 through October 2021 shows, only 464 of 2,303 abortions under the new law were performed on grounds of rape and a mere 125 seekers were under 18.^1^

The VTP Act introduced into Article 119 of the Health Code a new paragraph that requires teams of health workers to confirm the alleged facts and the gestational age, then advise the person seeking an abortion or her legal guardian (in case of girls under 14 years old or a woman with cognitive disability) and the area hospital within 24 h. If the seeker is under 14, rape is a statutory offense under Article 369 of the Criminal Code and hospital and clinic directors are required to notify the National Children’s Service and the Office of the Public Prosecutor to begin criminal proceedings. Prosecutors must also be notified if a victim is over 18 and the rape has not been reported so that criminal proceedings can be considered.

Under the comprehensive care and counseling guidelines issued by the Health Ministry for cases under the VTP Act, requests for abortion on grounds of rape must be substantiated by a secondary-tier facility^2^ such as a high obstetric risk clinic or similarly specialized unit, depending on the patient’s insurance coverage or the nature of the emergency (Government of Chile, Undersecretariat of Public Health, 2018, p. 77). Pregnancy termination itself is performed at a tertiary facility. The guidelines also require the primary tier to act as the point of entry for women requesting access to services under the VTP Act. The primary tier encompasses Family Health Centers, Family Health Community Centers, Primary Emergency Care Services, Rural Health Emergency Services, and Critical Care Emergency Services. These facilities are required to screen for and determine if grounds to suspect rape exist, offer patients accurate, timely and truthful information concerning pregnancy termination, and deliver support and counseling designed to help women disclose events of sexual violence (Government of Chile, Undersecretariat of Public Health, 2018, pp. 21–22).

This article presents our findings concerning rape cases, especially how survivors of sexual violence can turn into “ideal victims” in the eyes of medical practitioners. It reviews their gender biases, failure to screen for violence, and tendency to stigmatize women who request pregnancy termination –behaviors that can be exacerbated when involving some particularly vulnerable populations, including girls and women who are poor, homeless, migrant, or who abuse alcohol or drugs. The term *ideal victim* was coined by Nils Christie in 1986 to describe the stereotypical ways in which victim status is ascribed and how such stereotypes are legitimized. As Christie notes, the ideal victim is “[T]he tiny old woman who comes home in the middle of the day after caring for her sick sister. She is attacked by a man who hits her over the head and then steals her wallet to spend the money on drugs or alcohol” (pp. 276–277). To Christie, the discursive construction of the status of ideal victim has the following characteristics:

(1) The victim is weak. Sick, old or very young people are particularly likely to be ideal victims. (2) The victim was carrying out a respectable task -taking care of her sister. (3) She was where she could be without any inconvenience -on the street and in the daytime. (4) The criminal was large and mean. (5) The criminal was a stranger to her (1996, p. 277).

Stating that an “ideal victim” is the product of social and cultural stereotypes implies that the ways bodies and their sexual or gender assignments are understood belong within a certain point of view, historicity, and system of representation. The concept of gender, as expounded by Money (1995) in the biomedical sphere, was an analytical tool intended to understand sexual representations through rules, conventions, norms, and institutional practices linked to culture rather than to biology or to a predetermined nature. In this perspective, sex is a biological reality imposed at birth that culture can modify through gender (Money, 1995). To most feminists, the biological basis for sexual difference is not a product of nature on which a set of cultural practices are accommodated; rather, sexual differences themselves are a construct produced and developed by cultural practices; they are the effect of discursive precepts historically established through gender. As Butler points out: “Gender ought not to be construed as a stable identity or locus of agency from which various acts follow; rather, gender is an identity tenuously constituted in time, instituted in an exterior space through a stylized repetition of acts” (Butler, 2016). Gender is the cultural performance of reiterated acts that define the essence of being a woman or man –both the differences that divide the social corpus into sexes as well as the norms, attitudes, values and expectations that regulate the cultural repertoire of discursive identities, signs, and practices of males and females. As Lamas notes:

Gender roles are configured by norms and prescriptions dictated by society and culture regarding female or male behavior. Although these vary across cultures […] at their most basic, differences match primitive sexual divisions of labor: women give birth to and take care of children, therefore, femaleness is maternal and domestic while maleness is public. The male-female dichotomy, with its cultural variants (of the yin and yang type), creates stereotypes, more often than not rigid, that condition roles and limit human potential by stimulating or repressing behavior depending on gender appropriateness (Lamas, 2006, p. 36).

In Christie’s terms, “the ideal victim” is a privileged class of victim who is intersubjectively recognized on the basis of gender biases and stereotypes that impart legitimacy and create a reality. As Cook and Cusack (2010) explain, gender stereotypes are a set of beliefs about the characteristics of maleness and femaleness. “Beliefs can cover a range of components, including personality traits, behaviors and roles, physical characteristics and appearance, occupations, and assumptions about sexual orientation.” (p. 23). The gender attributes of an ideal victim –weakness, respectability, innocence– are assumed by social norms and significations in such a way that culture itself strengthens and repetitively idealizes them as the essence of maleness or femaleness; as a truth prior or external to the cultural construction of sexual difference. The ideal victim is thus an instrument for discrimination for both the dominant modes of inclusion and legitimization and for delegitimization or withholding of victimhood. Anyone failing to meet certain real or imagined gendered norms and social expectations will find it particularly difficult to access the rights and protections of victim status. In this study, the objective was to inquire about the implementation of the abortion law, the flow of care and referrals for women and to identify the main difficulties and strengths in access to services. The results revealed that the notion of “ideal victim” emerged time and again. Below we explain the research methodology and our findings.

## Methodology

This article summarizes an exploratory, qualitative study that examined health issues based on the experiences and reports of key actors. The researchers, once situated within the context that created the phenomenon (March et al., 1999; Amezcua and Gálvez, 2002), collected relevant data through 19 semi-structured interviews with a cross-section of (a) Primary-care physicians and midwives; (b) Teams of social workers and psychologists -known as the psychosocial team- in secondary and tertiary care who have direct contact with primary-care health personnel for the VTP act cases; and (c) Two Health Ministry officials responsible for health system management. Units of analysis with a relevant professional role in voluntary pregnancy termination were identified through purposive, non-random, snowball sampling (Martínez-Salgado, 2012).^3^ All respondents practice in Metro Santiago, home to over two-fifths of the country’s population.^4^ Interview criteria were pre-tested for flexibility and adaptability as fieldwork required and the proposal was vetted by the Research Ethics Committee of the Law School at Diego Portales University.

Interviews were conducted from November 2019 through December 2020. Due to the SARS-CoV-2 pandemic, effective March 2020 all interviews were moved online or conducted over the telephone. The interview questionnaire was developed through a conceptual operationalization of the research objectives, identifying various dimensions, such as: implementation of the law, difficulties/barriers to access an abortion, screening sexual violence, early detection of situations covered under the law, patient care and referral, among others. In addition, we considered the outcomes of previous studies on the implementation of the law.

Field notes were complemented by audio recorded with explicit respondent consent and sufficiency of data was determined under saturation criteria (Martínez-Salgado, 2012, note 20). All data was then analyzed for semantic content (Amezcua and Gálvez, 2002, note 18) and coded in ATLAS.ti 8.0 software for ease of systematization. Transcripts were processed through inductive coding to identify the categories and subcategories that answered the research question and met the goals stated in the study’s theoretical framework (Palacios, 2012).

## Findings

### Stereotypes, biases, and stigma surrounding sexual violence in primary healthcare

The social and gender practices, beliefs and prescriptions produced by the discourse on sexual differences and gender roles were observed to have strong roots among primary health care practitioners. As a midwife noted,^5^ *“As in the past, women to them are not a whole complement or complete human beings. They’re just vaginas, tits, and pregnancies.”* Indeed, abortion seekers whose life experiences do not conform to dominant gender norms all too often face biases and stereotyping. As reported by one interviewee, women who request abortions often experience value judgments about their plight and are blamed for their pregnancies or for the sexual assaults they have endured:

*Midwives in primary care -not just because of lack of knowledge but also because of a tendency to blame women over sexual and reproductive health violence- […] are very judgmental of cases involving the [VTP Act]* (team member, social worker).

This is especially relevant where girls and teens are involved. A midwife reported having seen a 14-year-old who had become pregnant after “dating” a grown man, noting that some parents will normalize and overlook such relationships through stereotyped images of early sexual activity.

*The girl said she’d had her first sexual experience with a guy who was about 22-23. Her mother cried her eyes out, saying she just could not believe that her daughter had started having sex at such a young age […] but she then turned around and sort of normalized it by saying "My daughter also kind of likes fooling around and dressing suggestively. She loves acting grown-up* (team member, midwife).

As regards the mandatory reporting of sex offenses, this midwife also noted that some police officers will replicate stereotypes about a teen’s sexual experience, even presuming sexual consent if she is seen to be leading a “licentious life”: *“A cop said something like ‘Why bother? The girl’s going to get called in to make a statement, maybe they’ll ask you to give one, the girl will say it was consensual and that’ll be it, case closed’.”* Despite these stereotypical responses, the midwife said that the key reason to file a report is a perceived need to avoid liability, regardless of a patient’s claims of consent:

*The parents didn't want me to file a report because they just wanted the guy to go away. They said they’d look after their girl and her baby. But as a midwife working with sexually active teens, whether I want it or not, I have to report in order to protect myself […] I have to, whether the mother or the girl agree* (midwife).

Stereotypes about motherhood as the quintessential female identity also have a direct impact on the care being provided, regardless of age. Another midwife cited cases of 14-year-olds impregnated by their teenage boyfriends. Without inquiring too much into the context, she felt that these pregnancies were wanted: *“These girls have an option to claim the third ground [*i.e.*, rape] but do not, because in fact they and their boyfriends planned to get pregnant and are very happy about it.”* Another midwife concurred*: “We’ve had 12-year-olds who have had a baby and are happy about it […] They meant to become mothers* (midwife)”.

To these midwives, a presumed yearning for motherhood seemed like an adequate proxy for consent. But such views, in addition to potentially covering up instances of imposed motherhood, stigmatize girls who do not conform to expected patterns of sexual behavior. As one of the midwives noted about her health team: *“Where minors are involved, my colleagues do not really broach these subjects. To them it’s ‘all right, you wanted it so it wasn’t rape. Perfect’. It’s like ‘you asked for it’.”* Biases and stereotypes on wanted or accepted motherhood may also result in the services contemplated in the voluntary pregnancy termination law being held back. A health team member relates:

*When the hospital started to implement the law, it organized training sessions. At one such session we heard things like “A while ago we saw this girl who was pregnant as a result of rape but wanted to keep her baby, so we didn’t report it.”* (team member, social worker).

When pregnancy results from rape, the guidelines on comprehensive care and counseling require health institutions to provide biopsychosocial care, even if a woman has chosen to keep the baby (Government of Chile, Undersecretariat of Public Health, 2018, p. 83). But biases and stereotypes on teenage pregnancy, often compounded by inadequate practitioner training and unfamiliarity with the law, may result in noncompliance with the guidelines:

*Health care providers widely believe that all rape cases under the law should lead to pregnancy termination; conversely, women who choose to continue their pregnancies are not VTP cases. In their view, rape equals women who don’t want to be pregnant. That’s the logic* (team member, social worker).

Abortion-related stigma also varies depending on urban or rural status. As one respondent noted, greater access to goods, services and resources can help city dwellers make more informed decisions: *“They’re more empowered when it comes to their rights.”* The very socio-spatial configuration of urban areas enables greater anonymity and prevents isolation and exclusion by home communities. This stands as a far cry from the reality facing women in outlying or rural areas, where sexual violence is more likely to be hushed or covered up outright:

*[In rural areas] sexual violence in the home may not be more prevalent but it is more often swept under the rug. As such, if you don’t see too many cases, you wonder why. We know there’s a world of things we don’t hear about but do happen out there* (team member, psychologist).

### Mistrust and presumption of sexual consent

#### Women over 14

Interviews showed that gender biases and stereotypes can greatly impact access to services under the voluntary pregnancy termination law. Among some primary health care providers, institutionalized cultural patterns can lead to value judgments on women’s characteristics, conduct and lifestyle, often resulting in dismissal of their accounts and in a tacit shifting of blame onto them. Key factors at play include dress, sexual conduct, seemingly reckless exposure to risk, socio-economic status, alcohol or drug use, and kinship with offenders.

##### Vulnerability as exposure to sexual violence and to labelling as an ideal victim

One health team member spoke to the credibility issues facing homeless alcohol or drug users. Her account illustrated how such women would not be regarded as an ideal victim in terms of accessing the abortion law:

The attending midwife verified that the patient was pregnant. She cried and cried but said nothing. The midwife then told her: “You’re using [drugs] and you live on the street. You need to be seen by a social worker. Just wait here.” Once left alone, the patient slipped out. At no point did the midwife consider possible that the woman might have been raped (team member, social worker).

According to this respondent, the midwife not only failed to check if pregnancy was the result of rape; she also offered no guidance, no emotional support, and no information on the services contemplated in the abortion law. The respondent noted that such attitudes are not rare among some primary sector midwives. In another case, although rape had already been established at the secondary tier, when informing the primary health center by e-mail *“The attending midwife (…) warned that since these patients will say anything, we were not to just go ahead and believe them.”* Poverty and irregular migration status only add to vulnerability. As a midwife pointed out, poor or migrant women are particularly exposed to sexual violence or to being coerced into sex.

*Poor women are more likely to be sexual assault victims. A woman who can’t afford the bus fare will be forced to walk alone at night; a woman who needs a job or a place to live may be forced to agree to certain conditions […] We see a significant share of migrants. We can only conjecture, but generally, being an illegal migrant or working low-paying jobs unfortunately places them at greater risk for sexual assault* (midwife).

A midwife recounts the case of a migrant woman who asked to terminate a pregnancy that resulted from having been raped at a social gathering: *“She went to this party, had too much to drink, and woke up being groped by a guy she did not know”* (midwife).

##### The plausibility of accounts

As the Health Ministry guidelines indicate, a crucial component of establishing the crime of rape is ascertaining the plausibility of victim accounts. *“We assess their bona fides […] We conduct interviews and determine whether grounds exist to suspect the crime of rape”* (team member, social worker). This raises questions about how to construe the plausibility test and the determination criteria used. Under the guidelines, in order to find whether events could constitute the crime of rape, health teams must review the person’s accounts, notably the aptness of events to result in pregnancy and the correspondence between gestational age and the reported date of rape (Government of Chile, Undersecretariat of Public Health, 2018, p. 82). A team member explained that this step only entails taking the victim’s statement and determining gestational age. To avoid revictimization, it does not involve judging the accuracy of the facts.

*The interviews we conduct are only meant to determine the gestational age and whether events could constitute rape […] We are not judges and don’t much believe in so-called medical committees […] It is quite intimidating already for women to have to convince three, four or five people of their stories, so we try to have a conversation in a safe, welcoming environment* (team member, psychologist).

Other respondents agreed that it is not their job to verify the truth of women’s accounts before the crime of rape can be established:

*As long as I’m able to provide the plausibility opinion I’m required to render, I just try to take down unprompted accounts instead of cross-examining women or delving too much into side events. Our job basically involves making a simple, broad-based judgment call; these aren’t reports about extraterrestrials landing on Earth* (team member, psychologist).

Another health team member explains: *“The third ground [rape] requires accounts to be consistent with clinical findings,* i.e.*, the event happened so many weeks ago and I’m so many weeks pregnant. If it is a consistent account, rape is judged to be likely.”* That said, inconsistencies about the last menstrual period may sometimes cast doubt on victim accounts. *“Lots of questions can arise if a woman is unclear about her last menstrual period. In such cases dates need to be either approximated or based on an ultrasound”* (team member, social worker).

##### Victim credibility: The legitimizing role of filing a report

The voluntary pregnancy termination law requires public hospitals and private clinics to notify public prosecutors so proceedings against a suspect can be started (Health Code, art. 119(2)). The Act does not mandate reporting by the survivor for the crime of rape to be established. A team member noted that most women requesting pregnancy termination do not previously file a report with police or prosecutors: *“Most women 18 and up hardly ever do.”* Another added that some prosecutors will require a written victim statement, even if she will not press charges:

*A chief prosecutor who is very interested in these cases decided to use a statement format which is very similar to a police report […] He practically wanted victims to name the suspect and all that, but so far we’ve been able to steer clear of this requirement. But he did ask every hospital in the area to use the format* (team member, psychologist).

Clearly misinterpreting the VTP Act, some medical practitioners hold filing a report to be a precondition for accessing services under the law. Such unfamiliarity with the law often leads to needless exertions to validate victim accounts, including third-party referrals that only subject women to secondary victimization. Says one midwife: *“We explain everything to the girls, including that we are required to report the incident because of their age. We ask if it was really rape and if so, whether they agree to report it themselves. If they refuse, we report nonetheless.”* A primary-tier physician recalls the arduous journey a pregnant woman with disabilities had to undertake to prove she had been raped:

The attending Ob-Gyn at the primary health clinic quite incorrectly told her she first needed to file a report. I can’t recall if the woman went to the police first and then to the prosecutor’s office or the other way around, but in short, she did both. Then a prosecutor sent her to the Forensic Medicine Institute for confirmation and a finding of rape. Starting at around 11 a.m., she spent the day going from the health clinic to the police, then to the prosecutor’s office, and finally to the forensic institute before the crime of rape could be established, which finally took place at around 9 p.m. She immediately went to her local hospital […] where the medical team on duty went over her account and the legal steps she had completed, and agreed that the crime of rape had been established (Doctor).

#### Girls under 14

The accounts of especially vulnerable girls and teens are also often dismissed. A midwife cited the case of a homeless, pregnant 14-year-old who *“…had partied and used drugs with other teens. She had totally blacked out and did not recall who or how many she’d had sex with.”* When the girl walked in she was 20 weeks’ pregnant, beyond the 14-week time limit set in the VTP Act (Health Code, art. 119). But instead of asking whether the pregnancy was the result of forced sex, the midwife chose to cast doubt on the facts provided by the girl because of her socio-economic status and sexual conduct: *“That’s what she says. Truth be told, we do not know what really happened.”* To avoid being stigmatized by medical practitioners, victims will often deny the absence of consent. A health team reported the case of a 14-year-old who had been raped at a party, but after persistent grilling from a midwife, had changed her version of events and eventually retracted the request:

*At the height of the pandemic I saw this 14-year-old girl […] who caught my eye: 14 and pregnant, that’s statutory rape […] So, first the attending midwife talked to her. The girl said she’d been raped at a party where there was a lot of drug use. She was so out of it, she did not remember how many guys she had had sex with. […] According to her, everyone at the party was underage, but we obviously don’t know that for a fact. When we explained that we’d have to report her rape, she recoiled; she seemed to be covering for someone. Now, the big question was the gestational age, since based on the dates she provided, she was too far along. So, she started out by asking for an abortion, eventually switched her story and ended up saying she didn’t want one* (team member, psychologist).

Such outcomes also crop up when prosecution of the statutory rapist is a distinct possibility. Under Chilean criminal law, the pregnancy of a minor under 14 is always the result of statutory rape.^6^ Public hospitals and private clinics alike are required to report these cases to police or prosecutors and notify the National Children’s Service (Health Code, art. 119(2)). Yet, underage victims will often deny lack of consent in order to avoid a criminal investigation and shield culprits -usually their boyfriends- from prosecution. However, as the law states, as long as a victim meets the requirements, she has a right to terminate her pregnancy and consent or lack thereof is irrelevant. But some midwives do not question the specific context of the relationship or the boyfriend’s age –to them, just being in a relationship and having started sexual activity at an early age suffices to rule out a victim’s meeting the requirements of the law:

*We’ve seen pregnant girls whose grandmothers will tell you that they were sexually assaulted. As a midwife, if the girl is 12-13 years of age, if I see a pregnant girl of that age*, *I just have to report. But when the police arrive, since the girls realize the stakes, they will obviously protect their boyfriends. Then you meet the boyfriend and it becomes clear that the girl was never assaulted and that the grandmother passed it off as such because she wanted her granddaughter to have an abortion* (midwife).

Other midwives understand full well that, a consensual relationship notwithstanding, being under 14 and pregnant constitutes statutory rape:

*We take the girl’s medical history, we ask if she is in a relationship, whether she used protection, if she’s been abused or assaulted. We investigate to determine whether rape has occurred. But even if the sex was consensual, under 14 it’s still rape* (midwife).

## Intrafamilial sexual violence

The interviews also exposed the sexual violence being perpetrated by fathers, close relatives, and partners. In general, victims were reluctant to acknowledge the events or delve deeper into them. Respondents note that threats, shame, and fear of stigma or blaming help explain their reticence and need for secrecy:

*Most sexual abuse is committed by relatives or someone close, and keeping these events a family secret is established practice. Most such attacks […] generally by uncles, brothers, fathers or grandfathers, go unreported. They’re suppressed through fear and threats* (team member, social worker). *We’ve seen […] teens whose pregnancies we suspected as being the result of sex with a father, uncle, older cousin, etc. This sets in motion a protocol that begins with a home visit. Most girls are not forthcoming and will just say that the baby’s father has gone away and that’s it. If you get them a psychologist’s appointment, they’ll just clam up and refuse to discuss it* (midwife)*. This is very much like what we had when domestic violence programs were first getting underway. At first they didn’t have much of an impact because of shame and fear, but nowadays they’re much more consolidated. I hope that voluntary pregnancy termination programs are also heading that way* (team member, social worker).

Several respondents also noted that some medical practitioners do not regard forced sex with an intimate partner or close relative as sexual violence, and thus do not consider associated pregnancies to be the result of rape.

*It’s not like the girls are just coming in and saying “Hey, you know what, I need to terminate this pregnancy because it’s the result of domestic sexual violence”. Since most of the time [health teams] will question everything […] victims in such cases would tend not to be believed* (midwife). *Many cases of rape and non-consensual sex in the home go unreported. If every such case were taken seriously and we really tried to believe women rather than scrutinize their claims […] If we just went with what they need, many more of these cases would be reported […] But the people in charge will sometimes split hairs in order not to give the go-ahead […] In a perfect world, you should have someone listen to you and take you to a room where you can be safe… But in the real world, you are harshly judged* (midwife). *Although her boyfriend would beat and abuse her, this mother of four was glad she was pregnant again. She was in the early stages but she wasn’t going to do anything about it because it was his baby. Of course we see cases like that, cases of violence not just from boyfriends but also from the pregnant woman’s father. Although in the high-risk part of town where I work we see many such cases, this does not correlate with qualifying under the VTP Act or seeking an abortion* (midwife).

Shame and fear of not being believed will often lead to keeping rape-related pregnancies under wraps until after the 12-week time limit, when termination is no longer an option. *“This girl came in at 18 weeks […] saying ‘I was abused by my boyfriend’. But it was too late to do anything”* (midwife). To steer clear of being disbelieved or of the consequences of potential criminal proceedings, some women will conceal the lack of consent:

*A couple of months ago I referred this sexual violence case to a shelter. She went back to the guy two days later, even though she was pregnant and he was beating her black and blue. When I saw her next, she was visibly bruised. Women like that will deny everything and claim the sex was consensual: “I wanted to get pregnant. We’re happy”* (midwife). *Family Health Centers know that many domestic sexual violence cases go unreported [….] because women won’t talk and it’s hard to make them talk. If we were able to get at every actual case of violence the numbers would be much higher, but a lot of evidence on domestic violence gets missed […] Also, women need to voluntarily report but it’s hard for them to say “I need help”* (team member, psychologist).

A midwife believed that domestic sexual violence actually worsened during the COVID-19 pandemic and that many women delayed seeking help over health concerns:

*As domestic violence went up during pandemic lockdowns, rape and abuse within dysfunctional families probably increased as well. It can well be that girls who were raped by relatives did not seek help because they could not leave the house for months, so when many who became pregnant during this time finally walked into a clinic, there wasn’t much left to do. Human behavior can be unpredictable, but my impression is that this is what must have happened. Afraid of exposure to a new virus, women seem to have shied away from seeking help. Whatever the case, the fact is that pregnancies did rise considerably* (midwife).

Another midwife felt that inadequate screening for domestic sexual violence remains widespread in primary care, fueled by insufficient practitioner training and ineffectual victim counseling and support protocols:

*Hospital staff aren’t exactly caring. It’s not as if they’re going to sit down with a patient and say “Ok, sweetie, tell me what happened. Does your partner force you to have sex?” There isn’t much in the way of social or psychological support and some [health staff] won’t investigate at all* (midwife).

That said, other primary health care providers will still try to prevent revictimization by choosing not to refer women to outside health centers or providers:

*We have this long-time patient who, as we later discovered, was being sexually abused by her brother. She had already interrupted a pregnancy that was the result of a sexual attack by him. During intake she admitted that she already had a daughter by her brother. We chose not to refer her to legally-mandated counseling, because our experience with such patients is that they end up being shuttled back and forth from one psychologist to the next, having to recount their stories over and over again, which we feel amounts to excessive intervention […] Patients like that should stay with one psychologist instead of being referred and counter-referred all over the place. That’s not healthy* (team member, social worker).

## Discussion

Our investigation showed that treatment to rape survivors is often conditioned and mediated by discursive practices that give rise to the “ideal victim” stereotype, and in some cases, withhold “victim status” and hinder or prevent access to voluntary pregnancy termination. We also found that young women victim of sexual violence whose lifestyle or sexual conduct do not conform to gendered expectations of femaleness are judged or even dismissed as victims of rape. As Fohring (2018) notes:

This is exemplified by the young woman […] out drinking with friends, and therefore engaged in a not-so-respectable activity, who is assaulted by a partner or acquaintance —an offender who is neither big or bad, nor unknown to the victim. Contemporary discourse would also likely discuss the victim’s level of intoxication, apparel, sexual history […] and failure to fight back as additional reasons to incite blame or withhold victim status (p. 196).

As our interviews showed, suggestive attire, partying at night, using drugs or alcohol and teenage or premarital sex may lead to young women being considered “bad victims” (Tomasini and Morales, 2016). A bad reputation creates identities that depart from the ideals of femaleness and notions of demure young women under parental control. They may also undermine the credibility of sexual violence reports and reduce the chances of health teams finding the woman’s accounts plausible. Moreover, the stereotypes that set the normative conditions for victimhood may also extend to offenders: “The more ideal the offender, the more ideal the victim” (Christie, 2014, p. 283). An ideal offender is a burly, evil, unknown assailant who attacks a weak, respectable, innocent victim. Yet, in real life, most offenders are relatives, spouses, partners, friends, or acquaintances. Moreover, as several respondents noted, some practitioners will dismiss sexual violence and assume that all pregnancies occurring within a relationship are wanted. And especially if a young woman’s partner does not fit the description of the ideal offender, they will tend not to inquire much about sexual violence or consent in connection with pregnancies.

The gender stereotypes that result in the “ideal victim” also play a role in withholding victim status from adult women who undergo sexual violence in domestic or intimate relationship contexts. The Chilean Ministry of the Interior’s IV Survey on Domestic and Other Violence Against Women (Undersecretariat for Crime Prevention, 2020) shows that sexual violence in the home rose from 1.8 percent in 2012 to 2.8 percent in 2020. Some 2.2 percent of respondents reported being forced to have sex; 1.7 percent were physically forced to engage in sex; 1.1 percent were threatened by partners with withholding funds for household expenses unless they agreed to sex; 1 percent were forced to use no protection, and 0.8 percent were forced to give up contraception. Article 369 of the Chilean Criminal Code makes spousal rape a crime under the law, but as Christie notes, married women are sometimes not considered “ideal victims”: “When the man beat up his wife in my culture, and the police are called in, they called it, until recently, a case of ‘*husbråk*.’ That means noise in the house. Noise does not create good victims. Noise is something that needs to be muted. […] Wives are not ‘ideal victims.’ Not yet.” (2014, p. 278). Gender stereotypes also help create the symbolic mandate of the wife or lover who must be always willing and available. This notion, which remains strongly held in contemporary society, harks back to some extent to sexual activity being regarded as a marital duty. Article 102 of the Chilean Civil Code defines marriage as “…a solemn contract whereby a man and a woman join together in true and indissoluble wedlock, for as long as they live, for the purpose of living together, *procreating*, and assisting each other” (emphasis added), although article 33 of the new Civil Marriage Act does provide for suspension of the duties of cohabitation and fidelity after a legal separation. But if love and sex are the fundamental duties of marriage, it is argued, there can be no such thing as marital rape since lack of consent cannot be invoked in legally defining the act. As Randall points out:

Dominant legal images of ideal victims reveal myriad ways in which some women are almost automatically disqualified from the category of credible sexual assault victims […] the idea that men’s right to ongoing sexual access to their wives such that their female intimates’ consent to sex is, by definition, continuous, renders marital rape, by definition impossible (Randall, 2010, p. 409).

Randall further notes that stereotypes that presume the consent of sex workers also exclude them from victim status: “Women in the category of ‘wives’ and women in the category of ‘prostitutes’ -typically seen to be at opposites poles of so-called ‘respectability’- are often both assumed to be continuously consenting to sex. As a result of this assumed continuous consent, their claims to sexual assault are usually legally nullified (Miller and Schwartz, 1995).” In Chile, this very notion is contemplated in the last paragraph of Criminal Code article 369, which allows for partner or marital rape charges to be dropped at the victim’s request, unless a judge disagrees. This shows that women who endure sexual violence at the hands of their partners but do not fit the “ideal victim” mold may be refused the social and legal recognition normally accorded to victims. Moreover, as reported by survey respondents, some health care providers will not even acknowledge such attacks as rape (Stewart et al., 1996, p. 168). If practitioners do not see rape-related pregnancies as the result of a wider context of intimate partner violence, where consensual sex cannot easily be told apart from acts that wholly or partially lack consent, the standard of credibility required to establish the crime of rape will hardly be met at all.

Furthermore, while some women may lack the characteristics needed to be subsumed into the normative “ideal victim” model, others who are socially conditioned by forms of domination and exclusion that intersect gender, race, sex and class (Crenshaw, 1989) may not be acknowledged as victims at all. In Chile, Afro-descendant women –Haitian, Dominican, Venezuelan or Colombian migrants– constitute such an example of social and normative dismissal. Neither ideal nor real victims, these non-victims of sexual violence stand at the crossroads of gender, race, ethnicity, marginality and non-citizenship. As Long (2021) notes, “Some Black women (particularly those with an offending history), are not only non-ideal victims; their racialized construction as the ‘suspect’ is incongruous with the ideal victim and they are constructed as the ‘ideal offender’ (p. 14). Not only are they not ideal victims, they are not victims at all: they become the (un)victim.” While Afro-descendant migrants are often more vulnerable to sexual violence from their partners, those trying to enter the country illegally are additionally vulnerable to demands for sex from smugglers and to human trafficking for sexual exploitation purposes, both consensual and not (Aguilera et al., 2018).

In fact, if dominant discourses on gender, class, sexuality and race can succeed in producing and naturalizing certain cultural imaginaries that pass for a reality that is expressed in the status of “ideal victim,” it is because social norms, and the law in particular, will mask and even facilitate the work of stereotypes on maleness and femaleness. The notion of ideal victim resonates clearly in Smart’s “the gender of law” formulation. To traditional legal discourse holding the law as being wholly neutral in terms of the gender and sex of victims and offenders, Smart replies that stating that “the law is gendered” does not only mean that the law is sexist or male-oriented, but that it operates through discursive practices that regulate and distribute the norms of recognition on the basis of sexual difference. In fact, the law operates as the technology that produces a generic subjectivity based on certain false social and sexual stereotypes (Smart, 2000, p. 43). This logic helps understand why some non-ideal or non-victims are not only refused legal recognition, they are also blamed and even held responsible, as if they were the offenders. Not only are women denied survivor of sexual violence status over not conforming to the female ideal; the discourse on sexual difference being an existential, ontological and constituent condition of female subjectivity is reinforced. As Smart adds, “[I]n legal discourse the prostitute is constructed as the bad woman, but at the same time she epitomizes Woman in contradistinction to Man because she is what any woman could be and because she represents a deviousness and licentiousness arising from her (supposedly naturally given) bodily form, while the man remains innocuous.” (2000, pp. 43–44).

Survey respondents also noted that adult women tend not to report sexual violence. Their reasons include daunting degrees of red tape as well as fear of judgment, retaliation, or upsetting family relationships (Picasso, 2018). While Chilean law does not require a prosecutor or police report for the crime of rape to established, our interviews showed that some medical practitioners will use such a requirement as a proxy for credibility –the rationale being that a woman who is prepared to recount her story across multiple venues and institutions must be telling the truth. This practice, however, leads to re-victimization and constitutes a violation of women’s rights. Ministry guidelines stress that looking into the facts and ruling on the veracity of victim accounts is the job of courts and prosecutors; health care practitioners have no authority to do so (Government of Chile, Undersecretariat of Public Health, 2018, p. 82). Yet, the guidelines do not spell out the parameters for assessment or the scope of plausibility tests. The resulting leeway in establishing the crime of rape makes for a normative vacuum that is, however, legal and has the effect of introducing a degree of discretion into enforcement of the VTP Act.

Some studies have shown that adult women will not report sexual assault in order to avert the ‘victim’ label. Making reference to the 1992–2000 U.S. National Crime Victimization Survey, Weiss (2011) observes that “The perception of victims’ innocence and credibility may also impact the discretionary labels and punishments determined by criminal justice professionals” […] “Offenses may be seen as less serious and offenders less guilty when victims themselves are considered to have behaved in some manner that makes them less innocent in the eyes of the law (e.g., engaging in mutual combat or illegal activity, behaving recklessly.” (p. 451). This translates into 9 percent of victims of sexual violence choosing not to report to prevent being stereotyped as weak or fragile (p. 458). To address related stigma and blaming, some victimologists (Link and Phelan, 2001, p. 378) have proposed rethinking the category itself. Noting the philological, historical and philosophical definition of victim status (the term comes from the Latin *víctima*, meaning “destined for sacrifice”), Van Dijk remarks that “victim” is a semantically charged term that replicates and reinforces ideal victim stereotypes (Van Dijk, 2009, pp. 1–2). As Stanko also argues, “Creating a category ‘victim’ is one way of dealing with women’s experience of male violence. The role and status of ‘victim’ is separate from that of all women. ‘Victimism’, the practice of objectifying women’s experiences of male violence, serves to deny the commonality among sexually and/or physically assaulted women and their oneness with all women” (Stanko, 2014, p. 16). Victimologists and feminist movements alike would rather speak of ‘survivors’ (Clay-Warner and Edgemon, 2020), a term that de-emphasizes gendered expectations of the ideal victim –weakness, respectability, innocence– and stresses self-reliance, resilience, and strength. As Dunn puts it: “Calling battered woman ‘survivors’, while granting them agency, may only shift responsibility and attention back to them as individuals and away from the social structures and forces that they must overcome.” (Dunn, 2005, p. 23).

These findings shine a light on the gender stereotypes and biases permeating the habits, conduct, and thinking of Chilean primary health care practitioners in reference to women who become pregnant as a result of sexual violence. Their notions about the “ideal victim” hinder access to the rights, goods and services contemplated in the voluntary pregnancy termination law, to the detriment of real victims and of those women who may not be considered victims at all. Although the VTP Act decriminalized abortion in cases of rape, enforcement still revolves around culturally-based discourses and structures that must be dismantled if socially-shared, gendered notions of sexual violence are to be truly transformed. Governments that fail to take concrete steps to do away with such stereotypes and guarantee women’s rights only help sustain and further institutionalize discrimination.

Imposing on the health care system to report the rape when a woman request an abortion under the rape ground impinges upon woman’s right to privacy since not all women may want to make known the assault, the woman’s safety might be at stake if the agressor could retaliate especially if he is part of the family circle or related to her, and she is deprived of the right to autonomy on the decision to proceed with criminal charges. This requirement demands a normative change.

Health care practitioners must therefore be able to accurately identify women who have undergone sexual violence and offer pregnancy termination services as part of a process of psychosocial support that does not discriminate against or stigmatize their choices. This requires clear actions from central government and primary healthcare administrators to provide training on the law and its protocols, on gender violence and stereotypes. Practitioners should receive robust instruction and training designed to both increase their awareness of sexual violence and eradicate the obsolete gender roles and cultural ideals that operate as the normative model of femaleness. Sensitizing health professionals on sexual violence, and instructing on sexual and reproductive rights are necessary tools and minimum political and ethical obligations for the health system to change practices that violate women’s rights.

The government, for its part, should guarantee access to all services under the law that decriminalized pregnancy termination on three grounds so as to assure all women effective, timely, quality biomedical and psychosocial care.

This study is limited in scope as it was carried out in the Metropolitan Region of Santiago and did not include women nor girls to avoid victimization. Future studies should assess the situation in other regions of the country given their particularities: geography and sociocultural contexts revealing higher rates of violence against women and girls, and greater opposition of health care providers to legal abortion under the rape ground.

## Data availability statement

The original contributions presented in the study are included in the article/supplementary materials, further inquiries can be directed to the corresponding author.

## Ethics statement

The studies involving human participants were reviewed and approved by Comité de Ética en Investigación de la Facultad de Derecho, Universidad Diego Portales. The patients/participants provided their written informed consent to participate in this study.

## Author contributions

LC, AM, LV, NB, and JB participated in the coding process. JÁ and LC drafted the article. LC, LV, and AM designed the research. NB, LC, and LV conducted the interviews. All authors contributed to the article and approved the submitted version.

## Funding

The research received funding from Diego Portales University.

## Conflict of interest

The authors declare that the research was conducted in the absence of any commercial or financial relationships that could be construed as a potential conflict of interest.

## Publisher’s note

All claims expressed in this article are solely those of the authors and do not necessarily represent those of their affiliated organizations, or those of the publisher, the editors and the reviewers. Any product that may be evaluated in this article, or claim that may be made by its manufacturer, is not guaranteed or endorsed by the publisher.

## Footnotes

^1^See data set at https://www.minsal.cl/reporte-ley-ive/ .

^2^Chile has a tiered system of healthcare delivery encompassing private and public components. Public health operates under the National Health Service based on degree of complexity: (a) Primary outpatient care; (b) Secondary or specialized care); and (c) Tertiary care.

^3^Secondary and tertiary care teams of psychologists and social workers were deemed key informants as they interact directly with primary care and can offer an outside view of it.

^4^Population of Chile: 17,574,003. Population of Metro Santiago: 7,112,808 (40.5%; 2017 Census).

^5^In Chile, midwives are university-trained practitioners whose role includes providing sexual and reproductive health care to women throughout their life cycle.

^6^Under Criminal Code art. 362, vaginal, anal or oral intercourse with a minor under 14 constitutes rape. The specific intent is to safeguard the sexual autonomy and integrity of minor children deemed physically and emotionally unprepared to engage in sexual activity; guilt is determined solely on the basis of victim age and whether this fact was known to the assailant. It is not concerned with loss of consciousness, ability to fight back or abuse of a victim’s mental impairment or illness (these circumstances are addressed in art. 361 of the Criminal Code on rape of individuals 14 and up).
